# Crucial role of corticotropin-releasing hormone, corticotropin-releasing hormone -binding protein, mir-200c, and mir-181a in preterm delivery: A case-control study

**DOI:** 10.18502/ijrm.v21i9.14398

**Published:** 2023-10-30

**Authors:** Ehsan Mohiti Ardakani, Mahta Mazaheri, Ph.D., Mohsen Forouzanfar, Mahdieh Mojibian, Mojtaba Jafarinia

**Affiliations:** ^1^Department of Biology, Faculty of Science, Marvdasht Branch, Islamic Azad University, Marvdasht, Iran.; ^2^Department of Medical Genetics, School of Medicine, Shahid Sadoughi University of Medical Sciences, Yazd, Iran.; ^3^Mother and Newborn Health Research Center, Shahid Sadoughi University of Medical Sciences, Yazd, Iran.; ^4^Mojibian Hospital, Yazd, Iran.

**Keywords:** CRH, CRH-BP, mir-200c, mir-181a, Preterm labor.

## Abstract

**Background:**

Preterm birth before 37 ${}^{\text{th}}$ wk of gestation is called premature birth. Corticotropin-releasing hormone (CRH) and CRH-binding protein (BP) act on various maternal and fetal tissues during pregnancy, such as the myometrium, which regulates the transition from the dormant phase of the uterus to the active phase. Studies have shown that mir-200c and mir-181a interact with CRH and CRH-BP.

**Objective:**

The present study aimed to investigate the expression of mir-200c, mir-181a, CRH, and CRH-BP in women with a history of preterm birth.

**Materials and Methods:**

In this case-control study, the gene expression level of mir-200c, mir-181a, CRH, and CRH-BP in placental tissue samples obtained from 48 women with a history of preterm labor was assessed in the Mojibian hospital of Yazd, Iran, from January to March 2023. Differences between *mir-200c*, *mir-181a CRH*, and *CRH-BP* gene expressions among cases and controls were assessed.

**Results:**

The outcomes indicated that the expression of CRH increased with going on to the regular parturition time (p < 0.001). While outcomes indicated, CRH-BP decreased with going on to the regular parturition time (p < 0.001). In addition, the results showed that the expression of mir-181a increased and mir-200c decreased with approaching the normal delivery time (p < 0.001).

**Conclusion:**

In conclusion, the expressions of mir-200c, mir-181a, CRH, and CRH-BP were dissimilar in different weeks of gestation. It could be proposed to use mir-200c, mir-181a, CRH, and CRH-BP as biomarkers to weigh the exact delivery time, which could minimize the side effects of preterm labor for the mother and fetus.

## 1. Introduction

Premature birth, occurring before 37 ${}^{\text{th}}$ wk of gestational age, is a leading cause of infant mortality (1, 2). Therefore, reducing infant mortality rates requires identifying the causes of premature birth (3-5). Despite the advances in medical science and childcare that have increased the survival rates of premature babies, the number of premature births has not significantly decreased. Because multiple factors can cause preterm birth, it is crucial to identify these factors and screen them in pregnant women for safe delivery. This identification can increase their participation in effective screening without requiring invasive procedures that can lead to miscarriage. Using non-invasive biomarkers to diagnose the condition is, especially valuable for pregnant women.

MicroRNAs, small noncoding molecules, play a critical role in regulating the expression of various genes necessary for a successful pregnancy. Therefore, it can be argued that microRNAs could be noninvasive and an effective marker for predicting a successful pregnancy. Several studies have demonstrated the role of microRNAs in infertility, diabetes, pregnancy, and fetal development (6-8). During pregnancy, microRNAs are primarily responsible for maintaining uterine stability. However, they also play other important roles, such as promoting fetal angiogenesis, facilitating the development of placental cells, and contributing to fetal heart development (9-12).

The corticotropin-releasing hormone (CRH) encoded a peptide hormone, which secreted from the hypothalamus. It remarkably impacts vital bodily processes such as growth, immunity, and reproduction. CRH is also produced by the placenta and intrauterine tissues involved in the onset of labor (13-15). CRH acts during pregnancy on various maternal and fetal tissues, such as the myometrium, which regulates the transition from the dormant phase of the uterus to the active phase (16, 17). Membrane receptor-binding G proteins hold CRH-related activities in the myometrium (18). During gestation, specific messaging cascades can be activated by receptors. One such receptor is the CRH receptor, which can be interacted with by the CRH-binding protein (CRH-BP). The primary source of CRH-BP during pregnancy is the maternal liver (16, 17). CRH-BP can bind to CRH with similar or higher affinity than the CRH receptors, leading to deconformation of the protein and depleting CRH from circulation, thus preventing its role (18). The balance between CRH-BP and CRH is critical during pregnancy, especially in the last weeks, and a disruption of this balance can have irreversible consequences for both the mother and the fetus (17). A recent study has shown that mir-200c and mir-181a, which affect CRH and CRH-BP, play a crucial role in preterm and term labor (19). Based on the importance of CRH, CRH-BP and their related miRNAs, mir-200c, and mir-1812b, this study aimed to examine their expression in women with a history of preterm labor. It is necessary to mention that computational algorithms TargetScan (http://www.targetscan. org/), PicTar (http://pictar.bio.nyu.edu/), and miRanda (http://microrna.sanger.ac.uk/targets) were used for the selected Mirna.

## 2. Materials and Methods

### Participants 

This study enrolled 48 women referred to perinatal care at Mojibian hospital of Yazd, Iran, in a case-control design from January to March 2023. The participants were divided into 3 groups based on gestational age (n = 16/each), the early preterm group (before 34 ${}^{\text{th}}$ wk), the late preterm labor group (34-36 wk), and the term group (37-42 wk). Each group was further divided into 2 subgroups based on mode of delivery, normal vaginal delivery and cesarean section (CS). After delivery, a placental tissue sample of 0.1 cm^2^ was taken from all participants and washed with saline buffer before being transferred to liquid nitrogen.

### RNA extraction, cDNA synthesis and real-time quantitative reverse transcription polymerase chain reaction (PCR)

Total RNA, also the miRNAs, were isolated from placental tissue samples QIAamp RNA Blood Mini kit, and miRNeasy mini kit (QIAGEN, Germany) and measured with Nanodrop2000 spectrophotometer and validated by agarose gel electrophoresis. For *CRH* and *CRH-BP*, cDNAs were synthesized with Revert Aid First Strand cDNA Synthesis kit (Thermo Scientific) using oligo dT and random hexamer primers. For *mir-200c *and *mir-181a*, cDNA was synthesized via Bon-Mir RT kit (Bonyakhteh, Tehran, Iran) according to the manufacturer's instructions. Glyceraldehyde 3-phosphate dehydrogenase (GAPDH) and small nucleolar RNA, C/D box were used as reference genes for mRNAs and miRNAs. qRT-PCR was performed using the SYBR Green PCR kit (Yekta Tajhiz Azma kit, Iran, www.yaktatajhiz.com) on the ABI thermal cycler detection system with designing primers (Table I), and expression levels were assessed using 2 ${}^{\left(-\Delta \Delta ct\right)}$ .

**Table 1:** qPCR primers used in this study


**Gene**	**Primers (5 '→ 3 ' )**	**Product size (bp)**	**T ${}_{m}$ ( ${}^{\circ }$ C)**
***CRH***	F: CAACCTCAGCCGATTCTGAT
R: CTAAATGCAGAATCGTTTTGGC	187	59 60
***CRH-BP***	F: AATGAAGGTTGGCTGGTGAA
R: CGCAGTAATCAGAGTAACGCTGTTC	184	58 59
***GAPDH***	F: GCACCGTCAAGTTGAGAAC
R: GGATGCTAGGGATGATGTT	171	58 58
***mir-200c ***	F: CGUCUUACCCAGCAGUGUUUGG
R: *	- -
***mir-181a***	F: AACAUUCAACGCUGUCGGUGAGU
R: *	- -
qPCR: Quantitative polymerase chain reaction, CRH: Corticotropin-releasing hormone, CRH-BP: CRH-binding protein, GAPDH: Glyceraldehyde 3-phosphate dehydrogenase, bp: Base pairs, T ${}_{m}$ : Melting temperature, F: Forward, R: Reverse, *The manufacturer did not send us the reverse strand sequence			

### Ethical considerations

The Ethics Committee of Azad University of Yazd, Iran, approved the study proposal (Code: IR.IAU.YAZD.REC.1401.075). Informed consent was obtained from all participants before enrolling to the study.

### Statistical analysis

The results were verified as the mean ± SEM. Differences between the variables of groups were assessed using one-way ANOVA test followed by Dunnett's multiple comparisons post-test. The delta-delta method was used in this study, and the term group was considered as a control in figures. P-values < 0.05 were supposed to be statistically significant. Statistical analysis was implemented via GraphPad Prism 6 software.

## 3. Results

### Expression analysis of *mir-200c* and *mir-181a*

**Figure 1:**
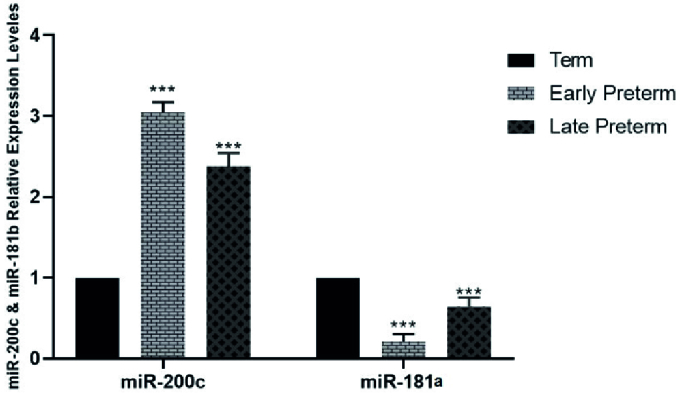
Comparison of the expression levels of *mir-200c *and* mir-181a *in normal vaginal delivery and term (control) groups via one-way ANOVA test followed by Dunnett's multiple comparisons post-test. ***P < 0.001.

**Figure 2:**
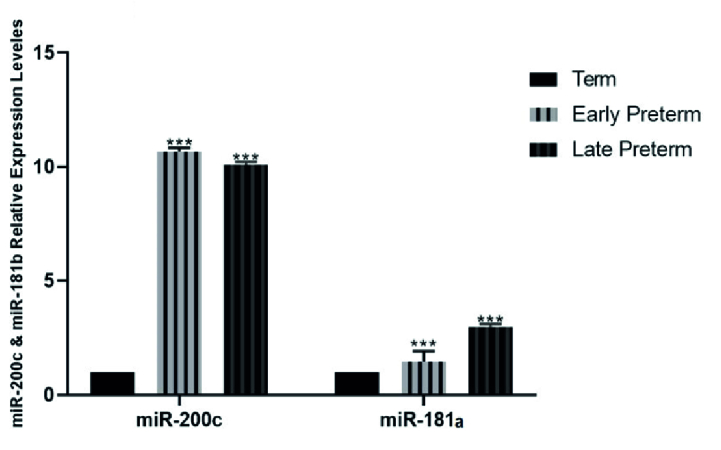
Comparison of the expression levels of *mir-200c* and *mir-181a* in placental preterm delivery and term (control) groups via one-way ANOVA test followed by Dunnett's multiple comparisons post-test. ***P < 0.001.

The study showed that mir-200c expression decreased in placental samples as regular parturition approached. Consequently, mir-200c expression was significantly higher in women with vaginal preterm delivery, particularly in the early preterm group ( < 34 wk) and the late preterm group (34-36 wk), compared to the term group (37-42 wk). CS outcomes were comparable to those of vaginal delivery. However, the expression of mir-181a in placental samples increased as regular parturition approached, and this was observed in both vaginal and cesarean delivery groups (Figures 1 and 2).

### Expression analysis of *CRH* and *CRH-BP*

**Figure 3:**
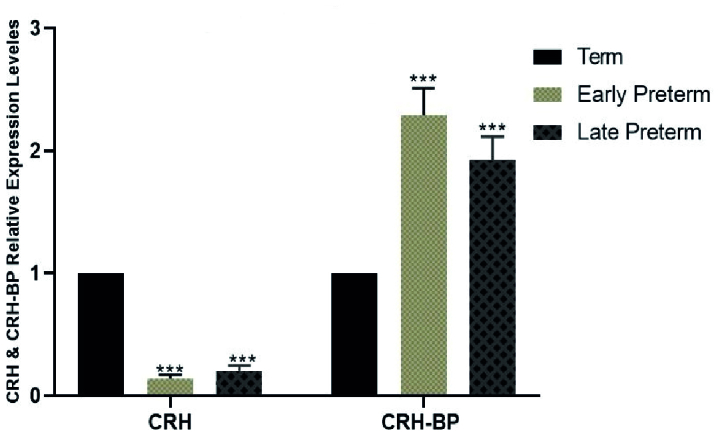
Comparison of *CRH* and *CRH-BP* expression levels in cesarean preterm delivery and term (control) groups via one-way ANOVA test followed by Dunnett's multiple comparisons post-test. ***P < 0.001.

**Figure 4:**
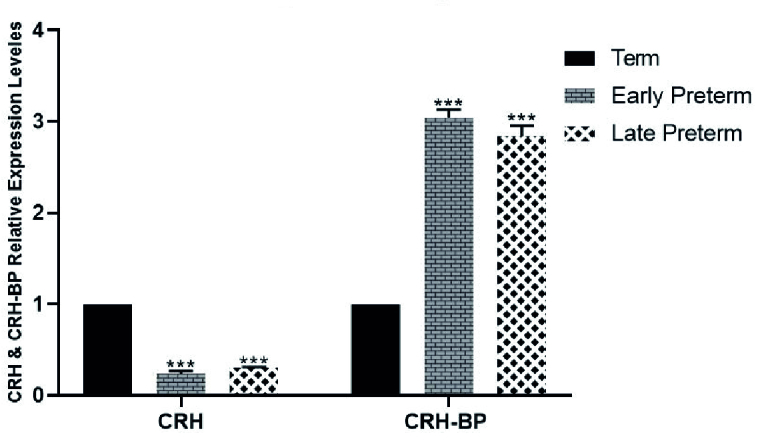
Comparison of *CRH* and *CRH-BP* expression levels in placental preterm delivery and term (control) groups via one-way ANOVA test followed by Dunnett's multiple comparisons post-test. ***P < 0.001.

Placental samples from women with vaginal preterm delivery and CS showed significantly lower expression of *CRH* in the early preterm group (before 34 ${}^{\text{th}}$ wk) and the late preterm labor group (34-36 wk) compared to the term group (37-42 wk). Conversely, *CRH-BP* expression in both vaginal and cesarean delivery groups decreased as the delivery time approached, as shown in figures 3 and 4.

## 4. Discussion

In the initial phase of this study, we examined the expressions of *CRH* and *CRH-BP* in our study groups. Our findings indicated that the expression of *CRH* increased as the natural parturition time approached. Specifically, we observed that women with vaginal preterm labor had lower *CRH* expression in the early preterm category (before the 34 ${}^{\text{th}}$ wk) and lower expression in the late preterm delivery group (34-36 wk) compared to the term group (37-42 wk). However, the expression of *CRH-BP* revealed different results. *CRH-BP* expression was nearly identical in the early and late preterm groups and significantly higher in the term delivery group. The outcomes of the CS group were comparable to those of the vaginal delivery group. We observed a slight increase in the CRH expression as the delivery time approached. In contrast, *CRH-BP* expression was notably decreased in the term delivery group compared to the early and late preterm groups. These findings suggest that the balance between the expression of *CRH* and *CRH-BP* genes is a crucial factor in the onset of labor.

In humans, CRH is involved in the mechanisms that control the onset of labor (14). Elevated maternal plasma CRH levels indicate progression from pregnancy to delivery (7, 20, 21). One of the target tissues for CRH is uterine muscle tissue. CRH prepares myometrial tissue for labor and possibly regulates uterine contractions during labor. Other target tissues for CRH include the placenta and embryonic tissue (22, 23). CRH acts as a fetal adrenal that produces cortisol and dehydroepiandrosterone sulfate (DHEA-S). DHEA-S is metabolized to estrogen in the placenta, increasing uterine contractions and facilitating labor (22). Early diagnosis of preterm birth allows the physician to prevent the consequences of preterm labor for the mother and fetus. Since remedies for preterm delivery have been less successful, much research is now focused on predicting preterm delivery. The process of preterm delivery is accompanied by physiological changes in the mother's body (23). These processes include lowering the mother's immune system to accept the fetus, becoming obese, and maintaining the womb's stability. For physiological changes and endometrial stability, modification in the expression of some genes is required. Some studies have indicated that abnormal gene expression might lead to infertility (24-27). In this work, we have endeavored to offer new results by investigating the expression of CRH and CRH-BP as well as mir-200c and mir-181a in tissues of the placenta of preterm labor. It was found that maternal plasma CRH levels increased during the final weeks of gestation, indicating the specific role of this hormone in the onset of labor, and also noted that the placenta is the major locus of expression of CRH and CRH-BP compared to myometrium tissues (17).

In addition, a study on the role of CRH in pregnancy and childbirth found that the placenta synthesizes CRH during pregnancy and releases it into the bloodstream at a considerable level, approximately 1-10,000 times greater than in non-pregnant women (13). Recently, CRH has been recognized as a regulator of glucose transporter proteins in fetal tissues, suggesting a link between CRH levels and fetal growth. These events indicate that CRH regulates birth timing by regulating the signal pathways that control myometrium contractions. In another section of this work, we valued the expression of mir-200c and mir-181a that interrupt CRH and CRH-BP expression. The results specified that the expression of mir-200c decreased with the ongoing regular parturition time, so the expression of mir-200c in women with natural vaginal preterm delivery was much higher in the early preterm group and was high in the late preterm group in comparison to the term group. While mir-181a expression analysis showed the expression of mir-181a increased, moving toward real-time delivery. The outcomes of the CS group were almost equal to the consequences of vaginal delivery.

Another study examining miRNAs and mRNA expression profiles in human placenta from preterm labor and preeclampsia concluded that placenta in preterm labor and preeclampsia show alterations in the expression of several miRNAs that could be effective in different stages of pregnancies (18). Augmentation of the CRH in pregnancy is associated with a decrease in CRH-BP. A decrease in CRH-BP during the last 3 wk of pregnancy results in a large amount of CRH, which is involved in the onset of labor. CRH can be used in the placenta as a pregnancy duration and delivery time marker. At the onset of labor, a rapid rise in CRH levels occurs and acts as the initiator of labor. CRH controls uterine contractions by directly and indirectly regulating the secretion of DHEA and prostaglandins. Augmentation of CRH levels in tissue samples occurs much faster in preterm labor than in normal labor. This early increase indicates that CRH, as a molecular clock, controls the length of pregnancy. In late pregnancy, high levels of CRH are likely to increase the expression of the contractile protein that initiates uterine contractions.

## 5. Conclusion

The finding of this study revealed that the expressive changes in mir-200c and mir-181a during the period approaching the normal time of delivery lead to an increase in the expression of the *CRH* gene and a decrease in the expression of the *CRH-BP* gene, due to the lack of expression difference in tissue samples, offered the usage of mir-200c, and mir-181a as biomarkers to evaluate precise delivery time, which could minimize the side effects of preterm labor for mother and fetus.

##  Conflict of Interest

The authors declare that there is no conflict of interest.
